# The form of uncertainty affects selection for social learning

**DOI:** 10.1017/ehs.2023.11

**Published:** 2023-05-22

**Authors:** Matthew A. Turner, Cristina Moya, Paul E. Smaldino, James Holland Jones

**Affiliations:** 1 Department of Earth System Science, Stanford University, Stanford, CA 94305 USA; 2 Division of Social Sciences, Stanford Doerr School of Sustainability, Stanford University, Stanford, CA 94305 USA; 3 Department of Anthropology, University of California at Davis, Davis, CA 95616 USA; 4 Cognitive and Information Sciences, University of California at Merced, Merced, CA 95340 USA; 5 Santa Fe Institute, Santa Fe, NM 87501 USA; 6 Center for Advanced Study in the Behavioral Sciences, Stanford University, Stanford, CA 94305 USA

**Keywords:** uncertainty, social learning, cultural evolution, agent-based modelling

## Abstract

Social learning is a critical adaptation for dealing with different forms of variability. Uncertainty is a severe form of variability where the space of possible decisions or probabilities of associated outcomes are unknown. We identified four theoretically important sources of uncertainty: temporal environmental variability; payoff ambiguity; selection-set size; and effective lifespan. When these combine, it is nearly impossible to fully learn about the environment. We develop an evolutionary agent-based model to test how each form of uncertainty affects the evolution of social learning. Agents perform one of several behaviours, modelled as a multi-armed bandit, to acquire payoffs. All agents learn about behavioural payoffs individually through an adaptive behaviour-choice model that uses a softmax decision rule. Use of vertical and oblique payoff-biased social learning evolved to serve as a scaffold for adaptive individual learning – they are not opposite strategies. Different types of uncertainty had varying effects. Temporal environmental variability suppressed social learning, whereas larger selection-set size promoted social learning, even when the environment changed frequently. Payoff ambiguity and lifespan interacted with other uncertainty parameters. This study begins to explain how social learning can predominate despite highly variable real-world environments when effective individual learning helps individuals recover from learning outdated social information.

## Graphical abstract

**Figure:**
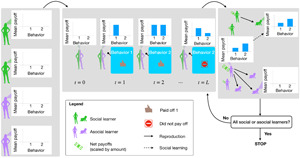

**Social media summary:** You won't believe this one weird adaptive trick social learners use … asocial learners hate it!

## 1. Introduction

Social learning enhances problem solving when acquiring information from others is more efficient than learning on one's own (Laland, 2004). However, social learning can also lead individuals astray if they are copying irrelevant, misleading or outdated information. Theoretical models have helped clarify the circumstances under which social learning should be evolutionarily favoured (Boyd & Richerson, 1985; Aoki & Feldman, 2014; Kendal et al., 2018), and these models have motivated empirical work that has both validated and refined theoretical predictions concerning the use of social learning in humans and other species (Galef & Laland, 2005; McElreath et al., 2005; Kendal et al., 2018; Allen, 2019). This literature indicates that social learning is a critical adaptation found across taxa for dealing with variable environments.

Uncertainty weighs particularly heavily on human adaptation because of our long lifespans, cosmopolitan distribution and dispersal across highly variable environments, requiring coarse-grained and plastic behavioural adaptations (Levins, 1962). However, the term ‘uncertainty’ is often used loosely in a way that fails to distinguish it from risk. *Risk* represents a decision with a variable payoff where the state space and probabilities associated with the different outcomes are both known. In contrast, *uncertainty* implies that the outcome probabilities, and possibly the state space, are not known (Knight, 1921; Keynes, 1921; Johnson et al., 2022). Furthermore, usage of the term uncertainty often conflates several different interpretations of that word. For example, different models define uncertainty in terms of the rate of environmental change, spatial environmental variation or the reliability of information acquired from the environment. As the number of formal models of social learning has expanded, an increasing number of modelling choices (Kendal et al., 2018) and formalizations of uncertainty have made it difficult to compare across models or to consolidate our understanding of the contexts in which social learning under uncertainty is adaptive.

Learning, social or otherwise, is an iterative process during which an individual acquires information, forms representations and predictions, and then tests and refines those representations and predictions to manage uncertainty (Jacobs & Kruschke, 2011; Clark, 2013). Because cultural evolutionary models are frequently designed to explain *social* learning mechanisms, they often contrast social learning with a simplistic mechanism for individual learning. In particular, a common assumption is that individual learners can pay a cost to definitively learn the optimal behaviour for a particular environment with certainty. This assumption implies that social learning, by observing an individual learner, is a good bet if the environment has not changed. Such modelling choices may lead us to overestimate both the extent to which individual learning provides quality information and the value of social learning in a population of individual learners.

To address these concerns we developed an evolutionary agent-based model that simultaneously operationalizes several forms of uncertainty and endows agents with a relatively powerful mechanism for individual learning. We model four kinds of uncertainty that are common in cultural evolution models and the related empirical literature: (1) temporal environmental variability; (2) selection-set size (the number of possible behaviours); (3) payoff ambiguity (the difference in the expected rewards for different behaviours); and (4) the effective lifespan of agents (the number of opportunities for individual learning). In our model, social learners learn from the previous generation and asocial learners do not. All agents engage in individual learning within their lifespans. The model also assumes that agents use an adaptive, empirically motivated learning mechanism, the softmax decision rule, which allows learners to make full use of the available data acquired by both individual and social learning (Sutton & Barto, 2018). Our model design thereby affords us a richer and possibly more accurate assessment of trade-offs between social and asocial learning. We use this integrated model to ask which kinds of uncertainty are likely to favour social learning and to clarify the logic underlying the results. In the remainder of this introduction we briefly review previous research on both the forms of uncertainty and the learning mechanism used in our model.

### 1.1. Varieties of uncertainty

Uncertainty involves a reduced ability to predict what will happen in the future or to assess which actions are likely to yield particular outcomes. Uncertainty can manifest in many ways. We focus on the following four sources of uncertainty for the evolution of social learning: (1) temporal environmental variability; (2) selection-set size; (3) payoff ambiguity; and (4) effective lifespan.

## Temporal environmental variability

When environmental variability is non-stationary (for example, because of climatic events or technological paradigm shifts), strategies that were previously adaptive may no longer be optimal. The difficulty in predicting either when such shocks will occur or which behaviours will be adaptive in the resulting environments leads to uncertainty.

Temporal environmental variability is fundamental to most evolutionary models of social learning. Such fluctuations have been proposed as an important selective pressure for learned as opposed to genetically fixed behaviours when genetic adaptation cannot keep pace with environmental change (Richerson & Boyd, 2000). On the other hand, environments that change *too* quickly will select against social learning so that individuals avoid learning outdated – and therefore maladaptive – information (Feldman et al., 1996; Boyd & Richerson, 1985). This suggests that intermediate levels of temporal variation are important for the evolution of social learning mechanisms (Aoki et al., 2005).

Temporal environmental variability tends to be modelled in one of several ways (Aoki & Feldman, 2014). Most commonly, it is modelled as an independent probability that the environment changes its state (and its corresponding optimal behaviour) before each new generation (Boyd & Richerson, 1985; Rogers, 1988; Feldman et al., 1996; McElreath et al., 2005; Enquist et al., 2007; Perreault et al., 2012; Aoki & Feldman, 2014), but it has also been modelled using deterministic cycles, so that environments repeat at regular intervals (Feldman et al., 1996; Aoki & Feldman, 2014). The consequences of environmental change can range from mild to catastrophic. In the latter case, a change of environment results in the total elimination of any adaptive behaviour, which must be learned *de novo* for the new environment (Rogers, 1988). Such catastrophic environmental changes present a large adaptive challenge as individuals cannot rely on accumulated information from previous generations. Other models of environmental change introduce less uncertainty as they fluctuate between two or more environmental states with corresponding adaptive behaviours. This means that previously maladaptive behaviours become adaptive when the environment changes (Perreault et al., 2012). The chosen mechanism for modelling temporal change has important theoretical consequences, reviewed in Aoki and Feldman (2014). Our study design ignores cases where temporal variation is low enough that genetic selection can canalize a behaviour, and instead considers only behaviours that can be learned.

## Selection-set size

When one does not know which option to take, uncertainty increases with the number of options. We call this selection-set size. In many studies of social learning, the selection set is often limited to just two options. For example, in empirical studies of bumble bees (*Bombus terresteris*) (Baracchi et al., 2018) and great tits (*Parus major*) (Aplin et al., 2017), experimenters provided two behaviours from which the bees or birds could choose, with one yielding a higher payoff than the other. Similarly, many human studies have used just two or three possible behaviours (McElreath et al., 2005; Morgan et al., 2012; Toyokawa et al., 2019). The designs of behavioural experiments are often motivated by modelling studies with similar formulations (Rogers, 1988; Boyd and Richerson, 1995; Feldman et al., 1996; Perreault et al., 2012). Larger, more open sets of behavioural choices are not uncommon in experiments trying to capture more complex or naturalistic tasks (Derex et al., 2013; Wasielewski, 2014), but these map imperfectly onto a theoretical literature that tends to use a limited set of behavioural options. Some models have studied systems with larger, but defined, selection sets (Rendell et al., 2010; Lindström et al., 2016), while in others the number of options is subsumed under the probability that a learner gets the right answer (Feldman et al., 1996; Enquist et al., 2007). Rarely is the size of the selection set explored explicitly as a source of uncertainty (although see Muthukrishna et al., 2016), even though the number of options one has is likely to increase the difficulty of the decision task (Haynes, 2009; White and Hoffrage, 2009).

## Payoff ambiguity

Most models of social learning necessarily differentiate between the payoffs for adopting optimal vs. non-optimal behaviours (Boyd & Richerson, 1985; Rogers, 1988; Enquist et al., 2007; Rendell et al., 2010; Aoki & Feldman, 2014). The size of the difference between these payoffs is usually taken to influence the strength of selection on learning strategies. However, the ability to discern payoff differences between behaviours is also a source of uncertainty. In reality, payoffs for particular behaviours are not always consistent. A behaviour may yield a large payoff sometimes and a small payoff other times (McElreath et al., 2005). This means that signals about the relationship between behaviour and payoff are often noisy, and differentiating between behavioural options is in part a problem of signal detection. When the difference in expected payoffs between optimal and non-optimal behaviours is very large, this noise matters little, as the signal is still very clear. However, when the expected payoffs of different behaviours are similar relative to the size of their variances, ambiguity arises about which behaviours really are superior. Smaller differences between payoffs corresponds to larger ambiguity. Payoff ambiguity has been manipulated in both theoretical (Perreault et al., 2012) and empirical (McElreath et al., 2005; Toyokawa et al., 2019) studies, both of which support the claim that payoff ambiguity increases the reliance on social information. Importantly, payoff ambiguity affects both uncertainty *and* the strength of selection, with smaller payoff differences leading to greater uncertainty for a learner and weaker evolutionary selection favouring optimal strategies. It is an outstanding problem to understand how payoff variance and payoff distributions generally affect evolved behavioural strategies (Haaland et al., 2019). For the sake of theoretical clarity for our goal of understanding the *interaction* of various sources of uncertainty, we only operationalize payoff ambiguity as the difference between expected payoffs, even though empirical studies have sometimes included both forms of payoff uncertainty (McElreath et al., 2005; Toyokawa et al., 2019), and a more complete model would include differences in mean and variance of payoffs.

## Effective lifespan

The more opportunities an agent has to learn during its lifespan, the more uncertainty can be reduced, assuming a stationary environment. Correspondingly, a reduction in the number of opportunities to learn will increase the uncertainty about which behavioural options are available and what their associated payoffs are. We refer to the number of individual learning opportunities within a generation as an individual's *effective* lifespan to highlight that it is the number of opportunities to learn about the payoffs associated with a behaviour, rather than the number of sunrises one experiences, that determines one's uncertainty about the behavioural options.

Empirically, the number of learning opportunities can be manipulated in the laboratory and in the real world will tend to correlate with an individual's relative age. In multi-round studies of information use in novel tasks, US participants’ use of social information declined precipitously across rounds (McElreath et al., 2005), suggesting they were more likely to use social information when they were most uncertain about the task early on. In a more naturalistic context, Aplin et al. (2017) found that younger great tits more readily used social information compared with older individuals, possibly because they had accrued less information via individual learning, and possibly because younger individuals have the most to gain (because of higher reproductive value) by switching to superior behavioural options. Cross-cultural studies have highlighted the importance of childhood as a phase of heavy social learning in humans (Reyes-García et al., 2016). Young children are more likely to acquire their beliefs and simple skills from their parents than are older children or adults, which is at least partly due to the differential knowledge accrual between young children and the older adults to whom they direct the most trust and attention (Kline et al., 2013). While these phenomena map imperfectly onto effective lifespan in our model, given social learning is only allowed intergenerationally, the relative number of individual to social learning opportunities, i.e. this model's version of effective lifespan, do vary across tasks, individuals and species, yet most models assume only one learning opportunity per generation (Boyd & Richerson, 1985; Feldman et al., 1996; Henrich & Boyd, 1998; Perreault et al., 2012). Some models do allow several cultural generations within one genetic generation (Enquist et al., 2007; Rendell et al., 2010; Lindström et al., 2016), but little formal theory explicitly examines the role of learning opportunities in the evolution of social learning.

### 1.2. Individual-level adaptations to uncertainty

While the cultural–evolution literature suggests that several forms of uncertainty have played a role in the evolution of social learning, other cognitive mechanisms have probably also evolved in response to uncertainty (Volz & Gigerenzer, 2012; Johnson et al., 2013; van den Berg & Wenseleers, 2018). Many of these are flexible learning mechanisms that do not require imitating the behaviours of others. For example, when faced with greater uncertainty, individuals may adopt more exploratory learning strategies and may even preferentially test behaviours with greater observed payoff variance (Wilson et al., 2014; Gershman, 2019).

Many models of social learning simplify by using minimally cognitive agents. For example, a common modelling strategy compares the payoffs of agents with different pure learning strategies (e.g. those who only engage in social learning vs. those who only engage in individual learning) while revealing nothing about the cognition underlying individuals’ decisions (Boyd & Richerson, 1985; Rogers, 1988; Aoki et al., 2005). This sort of behavioural gambit is common in evolutionary modelling, but has also been criticized as ‘blackboxing’ key cognitive processes that are important to cultural evolution (Heyes, 2016; Kendal et al., 2018). Some more complicated learning strategies that make use of both socially and individually learned information have been studied (Enquist et al., 2007; Perreault et al., 2012), including those that integrate cognitively plausible mechanisms such as reinforcement learning (Lindström et al., 2016). This approach, however, remains the exception rather than the norm. In order to understand how social learning evolves as an adaptation to uncertainty, we endowed agents with an adaptive cognition based on a softmax decision rule capable of integrating information from various sources to maximize payoffs (Gershman, 2019), described in more detail below.

## 2. Model

In our model we allow just one trait to evolve: social learning. All other parameters are exogenous. Our primary outcome measure is the observed frequency at which the social-learning trait fixates in simulated populations. We study how the social learning fixation frequency responds to each of the four varieties of uncertainty considered in this paper.

A population consists of *N* individuals, each of whom must decide which of *B* behaviours to perform at each time step within a generation consisting of *L* time steps. The behavioural selection set is a multi-armed bandit with *B* arms. That is, agents can pick one of *B* behaviours with random-valued payoffs (Sutton & Barto, 2018; McElreath et al., 2005; Steyvers et al., 2009; Rendell et al., 2010; Schulz & Gershman, 2019). In each generation, exactly behaviour is more likely to pay off than all the rest and is therefore optimal. Agents optimize their net payoffs over their lifespan by quickly learning which behaviour is optimal and performing it as often as possible within their lifespan. At the end of each generation, agents are selected with replacement to reproduce a new generation of *N* agents, with the probability of reproduction biased by net payoffs. Agents who inherit the social-learning trait learn about payoffs from the previous generation, while asocial learners begin life only knowing the number of behaviours the environment affords, but have no knowledge of behavioural payoffs.

### 2.1. Model environment and uncertainty

The structure of the environment is specified by several parameters that remain fixed throughout the course of a given simulation (Table 1). The environment affords *B* behaviours, where *B* is the selection-set size. Uncertainty increases with *B*. Each behaviour is indexed by *b* and yields a payoff of 1 with probability *π*_*b*_ and a payoff of 0 otherwise. As such, *π*_*b*_ is equivalently the expected payoff of behaviour *b*. At the beginning of each generation, one behaviour is optimal and yields expected payoff *π*_high_; the other *B* − 1 behaviours yield a lower payoff, *π*_low_. The payoff ambiguity is defined by *π*_high_ − *π*_low_. We fix *π*_high_ = 0.9 to be constant across all simulations, so payoff ambiguity is varied by changing the value of *π*_low_. In other words, payoff ambiguity, and therefore uncertainty, increases with *π*_low_. Agents have *L* time steps to act and learn asocially per generation (i.e. the effective lifespan). Uncertainty decreases with *L*. At the start of each generation a new optimal behaviour is selected at random from the set of all possible behaviours with probability *u* (the environmental variability), otherwise the optimal behaviour remains unchanged from the previous generation. Uncertainty increases with *u*.

**Table 1.** Environmental parameters. These include our four main uncertainty parameters under investigation; *π*_low_, *B*, *L*, and *u*. Bold indicates default value tested.

Symbol	Description	Values tested
*N*	Population size	50, 100, 200, **1000**
*π*_high_	Probability that the unique optimal behaviour pays off, 1	**0.9**
*π*_low_	Payoff ambiguity: probability of *B* − 1 non-optimal behaviours pays off, 1	0.1, 0.45, 0.8
*B*	Selection-set size: number of behaviour options	2, 4, 10
*L*	Effective lifespan: time steps per generation	1, *B*/2, *B*, 2*B*, 4*B*
*u*	Environmental variability: probability optimal behaviour changes between generations	0.0, 0.1, …, 1.0

### 2.2. Agents and learning

All agents learn individually over their lifespan, and those with the heritable social learner trait additionally use social information to constrain their individual learning. For simplicity, we refer to agents with the social learner trait as social learners. Social learners begin life with behavioural preferences based on information learned from an agent chosen from the previous generation. Asocial learners begin life with no behavioural preferences. Agent-level parameters are described in Table 2.

**Table 2.** Agent-level variables. The first four (*s*_*i*_, 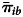, *c*_*ib*_ and *π*_*i*_) are dynamic with an implicit time dependence. The softmax greediness *β* and number of prospective teachers for social learners, *N*_*T*_, are constant throughout each simulation.

Attribute	Description	Initial value
*s*_*i*_	Social-learner trait: 1 if agent *i* is social learner; 0 otherwise	0 or 1 equally likely
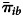	Mean payoffs acquired by agent *i* from behaviour *b*	*B*–vector of zeros
*c*_*ib*_	Count of how many times agent *i* performed behaviour *b*	*B*–vector of zeros
*π*_*i*_	Net payoffs accumulated by *i* within generation	0.0
*β*	Softmax greediness: ↑=more exploitation, ↓=more exploration	1, **10**, 100
*N*_*T*_	Number of teachers to pool, from which best selected	2, **5**, 10, 20

#### Individual learning

All agents perform individual, trial-and-error learning at each time step in their lifespan. Learning is guided by softmax search. Softmax search guides agents to exploit more frequently the most profitable behaviours when the agent is more certain it is the optimal one and to explore more frequently when the agent is unsure. To do softmax searching, agents track average payoffs acquired from each behaviour they have performed, which requires knowing the number of times they have performed each behaviour. The probability that an agent will perform a behaviour is a function of the agent's beliefs about a behaviour's average payoff in that time step and a fixed parameter that influences the amount of exploratory behaviour (more details below). The softmax function used here is a biologically plausible generalization of behaviour selection under uncertainty that enables agents to often greedily exploit the most lucrative behaviour they have observed, but also to sometimes explore alternatives (Yechiam & Busemeyer, 2005; Daw et al., 2006; Collins et al., 2013; Schulz & Gershman, 2019).

#### Social learning

At the beginning of each generation other than the first, each social learner selects one member of the previous generation to learn from in a payoff-biased way. A social learner selects this ‘teacher’ from the previous generation by first choosing the payoff maximizer among these (more details below). The social learner then inherits information about the likely payoffs of each behaviour from that one teacher, whereas asocial learners do not acquire this information. In this way, the social learner can potentially reduce the amount of exploration needed to both execute and learn about the optimal behaviour.

### 2.3. Dynamics

**Figure 1.**
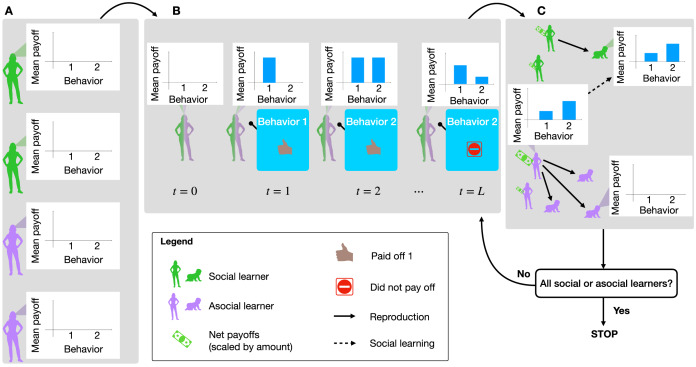
Model summary. Agents are randomly initialized as social learners or not, with their payoff observations all initialized to zero (a). Then agents begin selecting and performing behaviours and accumulating payoffs, which goes on for *L* timesteps (b). After *L* time steps, agents are selected to reproduce, social learner children learn from a member of their parent's generation, and the previous generation dies off (c). The simulation stops if children are all social or asocial learners (i.e. the system reaches fixation). Otherwise it repeats another generation and evolution continues.

Model dynamics proceed by first initializing the environment and agents according to the chosen parameter settings. Then, within each generation, agents select and perform behaviours, updating their estimated payoffs, and thus their probabilities of choosing each behaviour, along the way. Between generations, agents reproduce, teach social learners of the next generation, and die off. This process continues until the population has evolved to fixation as either all social learners or all asocial learners. Figure 1 illustrates this process.

#### Initialization

The model is initialized with *N* agents. Each agent *i* tracks observed mean payoffs for each behaviour, *b*, which is denoted 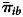. Each agent *i* also counts how many times they have tried each behaviour *b*, denoted *c*_*ib*_. At model initialization, 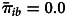 and *c*_*ib*_ = 0 for all *i* and *b*. One of the behaviours is chosen at random to yield expected payoff *π*_high_, with the other *B* − 1 behaviours yielding expected payoffs *π*_low_. Agents are independently randomly initialized with *s*_*i*_ ∈ 0, 1, so that ~50% of the initial population learn socially.

#### Within-generation dynamics

Within each generation, all *N* agents perform *L* behaviours sequentially and independently (Figure S3b). At each time step, agent *i* performs behaviour *b* with softmax-weighted probability:1
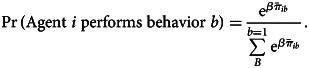
*β* adjusts how frequently agents perform behaviours with high expected payoffs (larger *β*) vs. how frequently agents explore alternative behaviours (smaller *β*). We used a default value of *β* = 10, but also varied this value in our sensitivity analyses (Figure S3).

Agent *i* using behaviour *b* receives a payoff of 1 with probability *π*_high_ if *b* is the optimal behaviour, or with probability *π*_low_ otherwise. After performing behaviour *b*, agent *i* updates its corresponding behaviour count for *b* by 1, 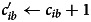. Then the observed average payoffs known by agent *i* for behaviour *b* are updated via moving arithmetic averaging,2
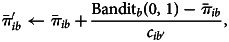
where Bandit_*b*_(0, 1) is the actual payoff received by agent *i* for behaviour *b* on a given time step. This form allows the running mean payoffs to be calculated for each behaviour, weighting each observation equally, without knowing in advance how many times a behaviour will be performed. While more or less sophisticated averaging may better represent specific species’ abilities in different contexts, this form provides a basic observational learning mechanism that may be further specified as appropriate for different cases.

#### Intergenerational dynamics

Between generations, agents first reproduce via asexual haploid reproduction, which determines the transmission of the social learning trait. Social learners then learn from the previous generation, after which all agents from the previous generation die off. More specifically, this all happens as follows. *N* reproducers are selected with replacement over *N* independent draws, biased by performance:3
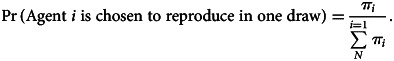


A child inherits its parent's social-learning trait *s*_*i*_ without mutation. A social learner child with *s*_*i*_ = 1 learns from a teacher from its parent's generation, including possibly their parent (i.e. both vertical and oblique learning are possible). A child chooses its teacher by first randomly selecting *N*_*T*_ prospective teachers from the population, then selecting the one in this set with the greatest payoff net, with ties broken randomly. By first subsetting *N*_*T*_ prospective teachers we represent the fact that access to the entire population is not generally guaranteed, though this type of algorithm yields qualitatively similar results to ones that do not first produce subsamples (Smaldino et al., 2019) and we show in the supplement that our results are robust to different *N*_*T*_ (Figure S2).

Social learner *i* adopts their chosen teacher *j*'s observed average payoffs for each behaviour, *b*, i.e., 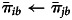; and agent *i*'s count of behavioural observations, *c*_*ib*_, is set to 1 if teacher *j* had at least one observation of behaviour *b*, and *c*_*ib*_ is set to 0 otherwise. This way, expected payoffs remain within [0, 1], but social learners are flexible to adjust to environmental change. Setting *c*_*ib*_ to 1 reflects the fact that the social learner treats the teacher's information as a single observation with the associated uncertainty, and serves as a useful baseline for understanding the interaction of different sources of uncertainty that we focus on here. In the real world this could represent the fact that some amount of information is always lost in direct instruction or other forms of social learning. If a learner's *c*_*ib*_ is set instead to the teacher's *c*_*jb*_, or some sizable fraction of it, learners will update their mean payoff values more slowly, making individual learning less adaptive. This could be desirable if there is good reason to have more confidence in a teacher's information, or there are other biological or cultural factors that make social learning more trustworthy. Asocial learners (*s*_*i*_ = 0) are initialized with *c*_*ib*_ = 0 and 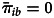 for each behaviour, *b*. The newly initialized population then repeats the within-generation dynamics.

After reproduction and die-off, the optimal behaviour changes with probability, *u*. The new behaviour is chosen from all other behaviours, with the current optimal behaviour excluded from selection.

#### Stopping condition

The evolutionary process continues until the population reaches evolutionary fixation with respect to social learning, that is, when 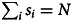 or 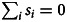.

### 2.4. Computational analyses

To analyse the effect of the four principal uncertainty factors, we systematically varied uncertainty values and observed how frequently social learning evolved across 1000 trial simulations for each combination of parameters tested. We observed three outcome measures (Table 3): first, the social learning (SL) fixation frequency, denoted 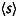, is how frequently social learning evolved to fixation across all trials for a given parameter setting. To support our analysis about why we observed the patterns in social learning fixation frequency that we did, we also measured the mean number of generations to fixation, which measures the strength of selection. Finally we measured the average net payoffs at the end of the simulation across agents and trials, normalized by lifespan. We analyse outcomes by plotting 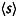 on the *y*-axis and environmental variability on x-axis, since we theoretically expect that 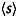 will always decrease monotonically from 1 to 0 as *u* increases.

**Table 3.** Outcome variables. All averages are computed across trials at the end of the last generation.

Symbol	Description	Values
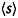	Social learning fixation frequency (Figure 2)	[0.0, 1.0]
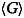	Mean time to fixation in units of generations (Figure 4)	{1,2,…}
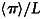	Mean payoffs accumulated across agents, normalized by lifespan (Figure 5)	[0.0, 1.0]

#### Implementation

Our model was implemented in the Julia programming language (Bezanson et al., 2017) using the Agents.jl agent-based modelling library (Datseris et al., 2022) and run on the Sherlock supercomputing cluster at Stanford University. All analysis figures except Figure 3 were made using the Julia plotting library Gadfly (Jones et al., 2021). Figure 3 was made in R (R Core Team, 2022) using the ggplot2 package (Wickham, 2016). Model and analysis code is publicly available on GitHub at https://github.com/mt-digital/UncMod.

## 3. Analysis

**Figure 2.**
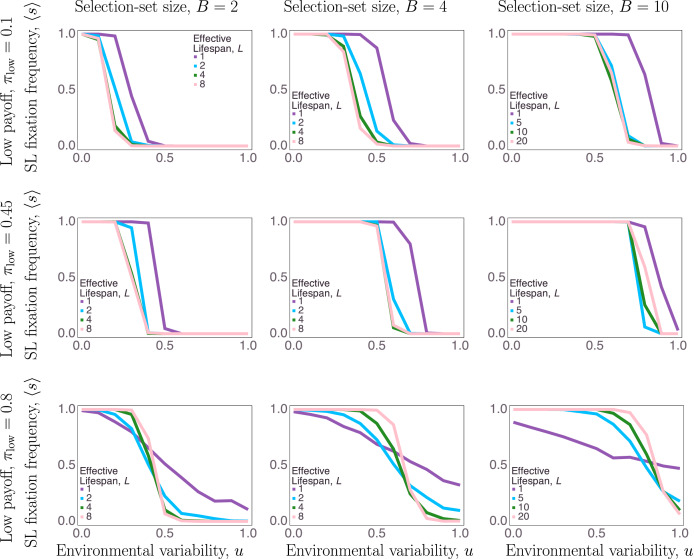
Effect of four forms of uncertainty on evolution of social learning. Social learning fixation frequency across trials (*y-*axes) monotonically decreases as environmental variability, *u*, increases (*x*-axes) in most uncertainty contexts. Other uncertainty values, payoff ambiguity, 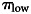 (rows), number of behavioural options, *B* (columns), and effective lifespan, *L* (keys), shift and flatten the slope from all-social-learner populations to all-asocial-learner populations.

Our main results are illustrated by Figure 2, which shows the proportion of simulation runs that fixated at 100% social learners as a function of each of our four uncertainty measures. First, we found that reliance on social learning monotonically decreases as temporal environmental variability, *u*, increases (Figure 2). When the environment is more likely to change between generations, information learned from the previous generation is less likely to be of value, decreasing selection for social learning. However, the exact nature of the relationship between social learning and temporal environmental variability was moderated by the other three uncertainty parameters.

**Figure 3.**
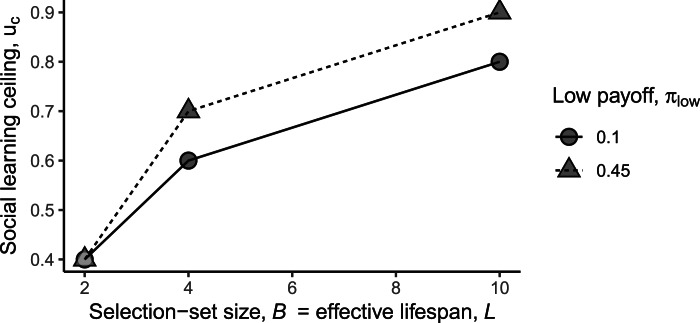
Effect of uncertainty on social learning ceiling. As the selection-set size, *B*, and effective lifespan, *L*, increase (set equal along *x*-axis), social learning frequently evolves across an increasingly large range of environmental variability values, i.e. up to increasingly large values of the social learning ceiling (*y*-axis; defined in Equation 4).

Across all parameter settings, we observed that there exists some threshold value of environmental variability beyond which the evolution of social learning is exceedingly rare. We call this the *social learning ceiling*, denoted *u*_c_ (Figure 3), which is expressed formally as:
4
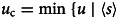

In other words, *u*_c_ is the smallest value of *u* for which social learning fixated less than one in 100 times. We will use this auxiliary measure to analyse the effect of the other uncertainty parameters and to highlight that social learning frequently evolves despite high levels of environmental variability.

The number of behavioural options, i.e., larger *B*, favours the evolution of social learning. Increasing the selection-set size expands the range of *u* over which social learning is favoured for two reasons. First, when the environment remains constant between generations, the value of social learning increases with more behavioural options, because it effectively increases the number of observations that one can learn from by comparing the payoffs of several models. In other words, relying *only* on one's individual trial-and-error learning is less likely to yield useful information when the search space is large. Second, when the environment changes, bias against the new optimal is weaker with more behavioural options. For example, with only two behavioural choices (*B* = 2), a common assumption in many models, social learning is particularly detrimental when the environment changes, since more of the population will have arrived at the optimal behaviour in the previous generation and socially learning from them will be biased against the correct choice. These two mechanisms make it particularly profitable to engage in social learning when the selection-set size is larger – for large values of *B* social learning can even be favoured when the environment is more likely to change than not (i.e., *u* > 0.5 – see Figure 3).

A shorter effective lifespan, *L*, also favours the evolution of social learning in most cases. When effective lifespans are long and there are many opportunities to gather information by individual learning, the value of social transmission is diminished. Given enough opportunities, all agents will learn the optimal behaviour eventually. Thus, under most conditions, longer effective lifespans lead to a narrower range of the parameters *u* and *B* under which social learning was favoured. It is noteworthy that in many cases, a large increase in the selection regime for social learning is observed when *L* = 1, and by definition no individual learning can occur. The exceptions to this general pattern occur for high levels of payoff ambiguity, *π*_low_ combined with low rates of environmental change, *u* (left parts of plots on bottom row of Figure 2). This reversal is partially explained by weaker selection when there is little difference in the payoffs between behaviours, and by the fact that the quality of social information is poorer when payoffs are ambiguous and lifespans are short (more on these effects of payoff ambiguity below). When *L* becomes roughly equal to or greater than *B* there is little marginal effect of increasing *L*, indicating that more individual learning experiences are unlikely to significantly affect an agent's ability to identify the optimal behaviour in their lifetime.

Of the four types of uncertainty studied in this paper, payoff ambiguity had the most complex effect on the evolution of social learning. Recall that we operationalized payoff ambiguity with *π*_low_, the expected payoff for non-optimal behaviours (equivalently the probability that those behaviours yielded a payoff of 1). This was relative to *π*_high_ = 0.9, the expected payoff for the optimal behaviour. When payoff ambiguity was low, non-optimal behaviours rarely paid off, so that exploration yielded reliable information and selection on learning strategies was strong. When payoff ambiguity is very high (*π*_low_ = 0.8), two things happen. First, agents are more likely to err by ascribing high value to non-optimal behaviours, since it is more difficult for them to discern the difference between optimal and suboptimal behaviours. Second, and perhaps more importantly, natural selection on strategies that do reliably select the optimal behaviour is relatively weak. This means selection is weaker in favour of social learning under low values of *u* and weaker against social learning under high values of *u* (Figure 2, bottom row). This effect was especially pronounced under other types of uncertainty, i.e. larger selection-set sizes and shorter effective lifespans – particularly when *L* = 1 where no individual learning occurred.

The effect of selection strength when the benefit of social learning is ambiguous is powerful and complex. We explain how the frequency of social learning evolves in such circumstances in the following section.

### 3.1. Strength of selection

To begin to confirm our interpretation of our analysis above, we measured strength of selection via the ensemble average of the number of time steps to fixation over all simulation trials for a given parameter setting. More steps to fixation here indicates weaker selection. Strength of selection is the extent to which the social-learning trait provided agents with a consistent benefit. Selection strength is minimal when social and asocial learning are equally beneficial. We denote the ensemble average over simultaion trials of the number of generations to fixation as 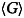, where fixation means that the number of social learners, 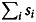, is equal to either zero or *N*.

**Figure 4.**
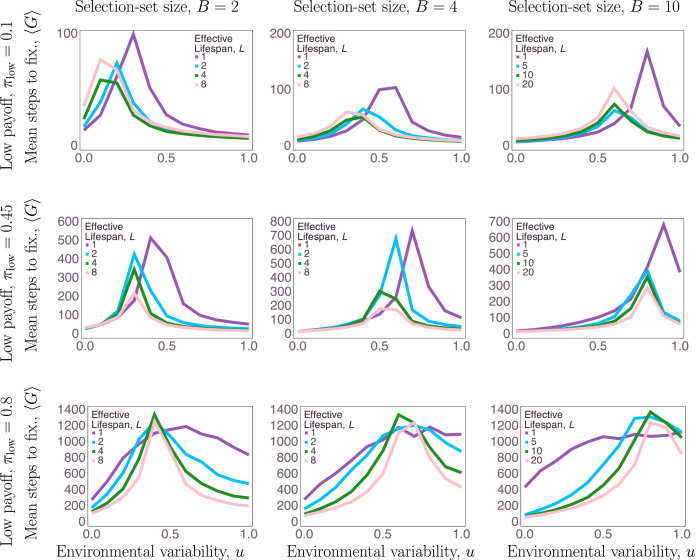
Average number of generations (*y*-axes) to fixation. Note that the *y*-axis scale varies between plots, indicating different overall time to fixation for different 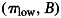 combinations.

Figure 4 shows the average number of generations to fixation across all uncertainty parameters. Strength of selection was weakest overall when payoff ambiguity was greatest, i.e. when *π*_low_ = 0.8. When payoffs are highly ambiguous there is relatively little advantage to the optimal learning strategy, and therefore relatively weak selective pressure.

The average number of steps to fixation peaks at environmental variabilities where social learning goes from being favoured to suppressed. Selection-set size, *B*, and payoff ambiguity, *π*_low_, had the clearest effects on which values of environmental variability maximized the number of steps to fixation. As selection-set size and uncertainty were increased, social learning was favoured for a greater range of environmental variability. Effective lifespan, *L*, had some effect on the location of peak time to fixation over environmental variability, but effective lifespan, *L*, had a more consistent effect on the overall selection strength: *L* = 1 trials took longer to reach fixation on average, especially when payoff ambiguity was low (*π*_low_ < 0.8). However, the effect of lifespan on time to fixation was non-monotonic – longer lifespans also resulted in longer times to fixation compared with intermediate lifespans (e.g. in Figure 4, top left and top right). When *L* = 1, selection pressure may be relatively weak since agents have no opportunity for individual learning, and social learning is the only learning channel. When *L* = 8 (*B* = 2, 4) or when *L* = 20 (*B* = 10), individual learning allows agents many opportunities to find the optimal behaviour, which means social learning provides a relatively weaker constraint on net payoffs over agent lifespans. This would also results in weaker selection pressure since there is relatively little difference between social and asocial constraints when *L* is large (Figure 4, top two rows).

### 3.2. Relative benefits of social learning in context

To check that populations evolved towards the learning strategy with higher expected payoffs, we also tracked the average net payoffs of agent populations across all uncertainty parameters and compared these with the payoffs of homogeneous populations of all-social learners or all-asocial learners. Average payoffs for the homogeneous populations were calculated by initializing simulation populations to be all-social or all-asocial learners, running the model for 100 generations so payoffs could stabilize, and finally averaging the net payoffs from the last generation across 1000 trials. Because simulations had no mutation, these homogeneous populations persisted as all-social or all-asocial learners.

**Figure 5.**
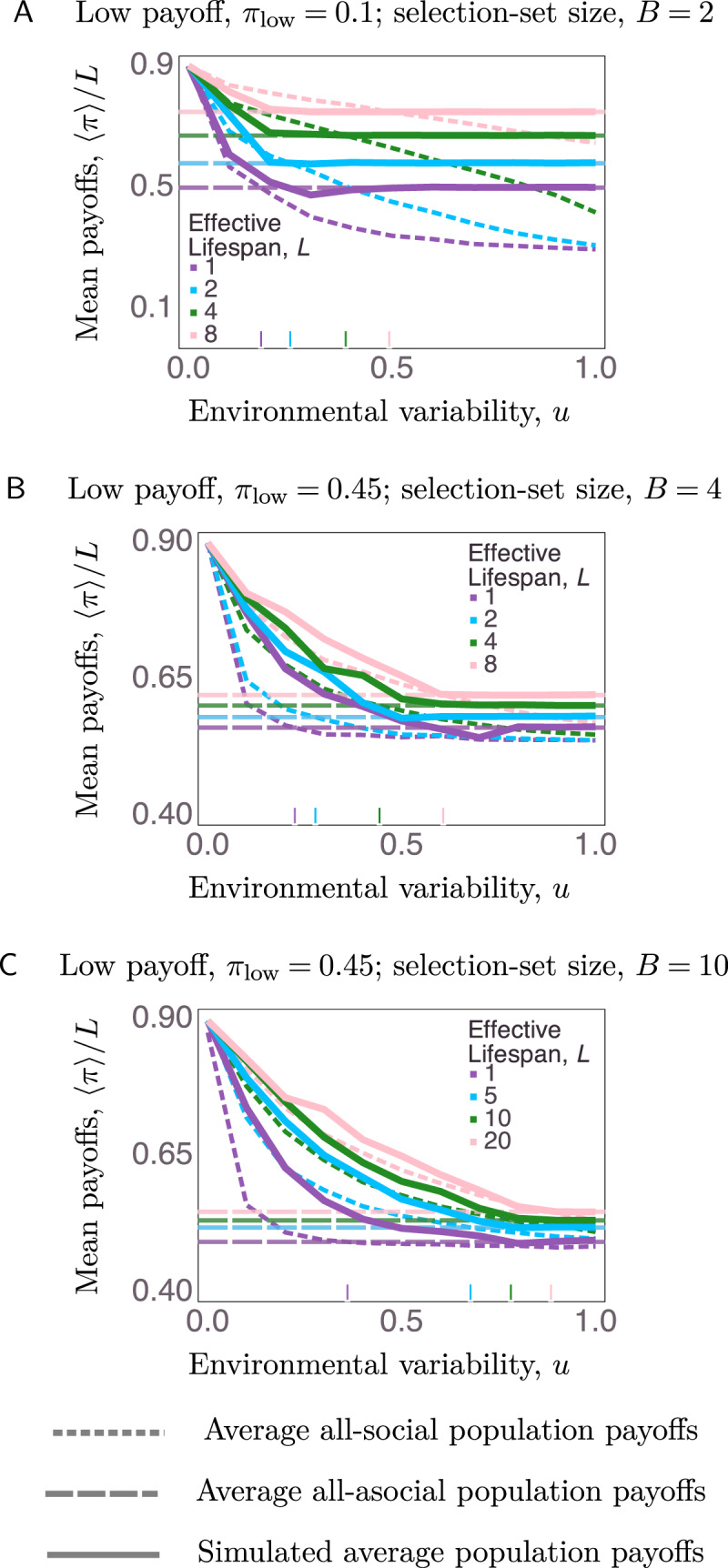
Comparison of observed average payoffs in simulations with average payoffs obtained by populations homogeneously initialized to be all social or all asocial learners. Often the simulated payoffs follow the payoffs from the better-performing homogeneous group, with some exceptions discussed in the main text.

Figure 5 shows the average net payoffs normalized by lifespan from a selection of our main simulations compared with the two reference homogeneous populations initialized either as all-social or all-asocial learners. Often the simulated payoffs follow the payoffs of the better-performing homogeneous group. However, there are several important deviations worth explaining. First, populations sometimes evolved to be asocial learners, even though all-social learner populations outperformed all-individual-learner populations when agents were long lived (*L* = 4 and *L* = 8) and in simple environments (*B* = 2; e.g. Figure 5A). Second, we also observed the opposite dynamic: agents from our main simulations who evolved to be social learners sometimes outperformed agents in the homogeneous social learner populations. This is most pronounced when *L* = 1, and even more so when *π*_low_ = 0.45 and *B* = 10 (Figure 5C). In fact, social learning evolved even in parts of the parameter space where the expected homogeneous social-learning payoff was lower than the expected homogeneous asocial-learning payoff (Figure 5B, e.g. *L* = 1).

**Figure 6.**
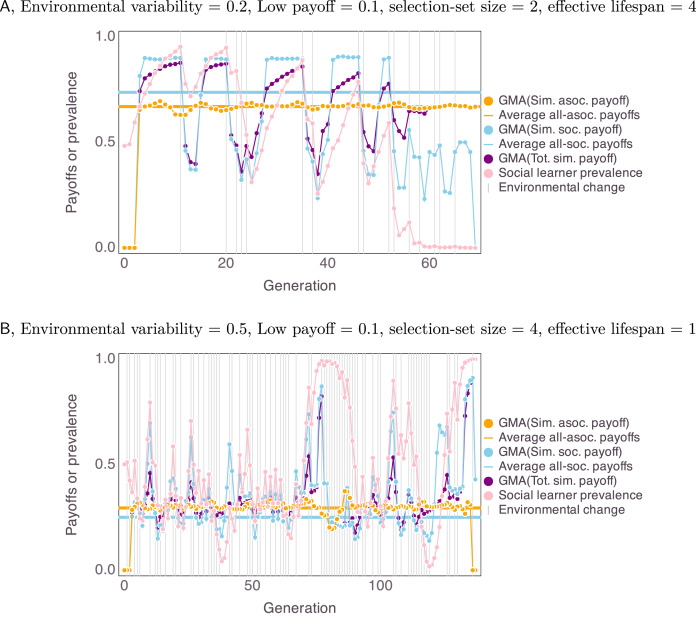
Example time series of geometric moving average (GMA) with windows of three payoffs from two select uncertainty settings (see main text), broken out by social learners, asocial learners and the whole population, compared with the expected homogeneous social and asocial population payoffs. Social-learner prevalence is also plotted. Vertical lines indicate environmental change.

Our simulations’ deviations from expected homogeneous payoffs reflect the effects of frequency-dependent payoffs and of stochastic environmental changes that affect geometric mean fitness. Frequency dependence is implicated in cases where our simulated agents that ended up as social learners outperformed homogeneous all-social-learner populations. In such cases, the earlier presence of asocial learners before social learning fixated enabled social learners in our simulations to outperform all-social learner populations. To illustrate the effects of stochastic runs of stable and unstable environments we inspected time series of individual model runs (Figure 6). We plotted the geometric moving averages (time window = 3) of the payoffs for the whole simulated population, and for the simulated sub-populations of asocial and social learners. We compared these mean-payoff series with the expected homogeneous social and asocial payoffs, the time series of social-learner prevalence, and to the timing of environmental changes within the simulation (Figure 6).

When payoff ambiguity and selection-set size were small (*π*_low_ = 0.1 and *B* = 2), and lifespan was long (*L* ≥ 4), asocial learning fixated because periods of extended environmental instability (see environmental changes after generation 50 in Figure 6a) caused social learners to more severely underperform compared to asocial learners, even though expected homogeneous social payoffs were greater than homogeneous asocial payoffs. Runs of environmental change strongly affected the geometric mean fitness of social learners who were weighing outdated information too heavily. In other words, environmental change lowers social learners’ mean payoffs, but social-learner payoffs build back up as the information quality in the population improves (blue dots, Figure 6a). Even though a homogenous population of social learners would outperform asocial learners in this case, unpredictable environmental stochasticity undermines the reliability of social learning. When lifespan was minimal (*L* = 1) and environmental variability was moderate (*u* ≈ 0.5), the opposite, unexpected outcome could occur where social learning fixates despite the fact that a homogenous population of asocial learners would outperform a homogenous population of social learners (Figure 6b). In this case, it takes relatively few consecutive generations with environmental stability to attract the population to all become social learners since social learners greatly outperform asocial learners during longer periods of environmental stability.

### 3.3. Sensitivity analyses

We performed sensitivity analyses to ensure that our main analysis was reasonably robust to a range of auxiliary parameters. These sensitivity analyses are presented in full in the supplemental material. First, we confirmed that our results were robust to various population sizes *N*, though smaller-*N* simulations demonstrated weaker selection strength, as expected, due to stronger effects of finite population size (Figure S1). Next, we verified model robustness to the number of prospective teachers, *N*_*T*_ ∈ {2, 20}, to supplement the main results that used *N*_*T*_ = 5. The number of prospective teachers did not change the results (Figure S2), except to increase time to fixation when *N*_*T*_ = 2, since the benefit of social learning may be more difficult to detect with fewer prospective teachers (Figure S2a). Finally, we confirmed our results were reasonably robust to a wide range of softmax greediness parameters, *β* ∈ {1, 100}, to supplement the main results that used *β* = 10. However, when *β* = 1, an insufficient reliance on the best-paying behaviour (i.e. low greed) caused agents to often ignore helpful social information (Figure S3a). On the other hand, too much greed (*β* = 100) made social learners inflexible so they could not recover as well when when they learned outdated social information. This cost to greediness was most pronounced for longer-lived agents who may spend several time steps performing a sub-optimal behaviour (Figure S3b).

## 4. Discussion

Despite the common claim that copying others makes sense when one is uncertain, we find that different forms of uncertainty can either promote or impede the evolution of social learning. By disambiguating various formalizations of uncertainty in a single computational model, we have developed a more nuanced theoretical understanding of how uncertainty affects the evolution of social learning. We reproduced the well-known results that the uncertainty derived from a temporally variable environment can limit the evolution of social learning if environmental change is simply too frequent for intergenerational transmission to be of value. Importantly, we also showed that this relationship is affected by other sources of uncertainty. We found that larger sets of behavioural options, shorter effective lifespans, and increased payoff ambiguity could all favour the evolution of social learning. However, these effects were not always straightforward. Increased payoff ambiguity and decreased effective lifespan weakened selection strength (i.e. evolutionary uncertainty in a finite-size population). Not surprisingly, when the consequence of one's choice does not affect payoffs much, selection either for or against social learning is substantially weaker. More interestingly, selection is also weaker when the ability to learn individually is fraught with uncertainty. Our model demonstrated that social learning can act as a scaffold for adaptive individual learning. We endowed agents with a biologically plausible individual learning mechanism based on a softmax decision rule. This enabled agents to recover from the potential cost of receiving outdated information. This, in turn, enabled the evolution of social learning even when social information was likely to be outdated.

Our modelling results suggest, on the one hand, that it might sometimes be better to ignore social information from previous generations since our natural world is rapidly changing (e.g. IPCC, 2022). On the other hand, humans have increasingly more behavioural options (selection-set size) with high levels of uncertainty about which behaviours are most beneficial (payoff ambiguity), and in this case our model mostly predicts a greater prevalence of social learning. In general, detailed theoretical models like this are likely to be critical for understanding how humans might adapt to an uncertain and rapidly changing world.

Our model necessarily made simplifying assumptions. These were chosen judiciously to address the main question of how uncertainty affects the evolution of social learning. Two critical assumptions we made have important consequences for comparing our results with previous findings in the literature. First, we assumed all agents engage in individual learning. This means that individual learning is effectively free, though potentially inaccurate. Although producing new knowledge may be costly, many other adaptive problems take the form of essentially cost-free individual learning. For example, it is hard not to encode association between clouds and rain, between thirst and drinking water, or between friendship and warm laughter. If there were an exogenous cost of individual learning, this would likely increase the social learning ceiling across uncertainty parameter settings. This means that our model does not capture the information-scrounging rewards to individual learning that produce mixed equilibria in precursor models (Rogers, 1988). Furthermore, our model operationalizes environmental variability as fully stochastic in a finite population. This makes it exceedingly unlikely for a heterogeneous population to persist since chance runs of very stable environments will lead to the extinction of asocial learners, while runs of very unstable environments will lead to the extinction of social learners. This further differentiates our results from precursor models that allowed for persistent frequency dependence of social learning benefit (Boyd & Richerson, 1985; Rogers, 1988; Feldman et al., 1996). Second, we only allow for intergenerational social learning and environmental change. Within generations, agents can only learn individually. This assumption is consistent with previous modelling work starting at least from Rogers (1988), and therefore allows us to replicate the classic finding that if the environment changes too quickly, socially learned information may become outdated. Adding horizontal social learning between vertical/oblique learning in this model would probably expand the range of environmental variability over which social learning would evolve (Turner et al., 2022), i.e. horizontal learning would increase what we have called the social learning ceiling. However, depending on the setting, there may be little difference between horizontal and oblique learning, for example if the generations are purely cultural ‘generations’ where parents, and others of the parent's generation, may also be peers.

We could have made alternative choices for a range of other structural features, the future exploration of which will be important to the development of more parsimonious theories of social learning. For example, we only considered success-biased learning, while people may use conformity or other context biases to learn socially (Boyd & Richerson, 1985; Muthukrishna et al., 2016; Smaldino et al., 2018). This modelling choice maximizes the potential benefit to social learners, which suggests that other mechanisms, like conformity, may lead to weaker selection overall. Furthermore, our social learning procedure could be interpreted as direct copying of latent psychological variables (expected payoffs for each behaviour). Such detailed information is most likely transmitted through teaching wherein the model explicitly informs the learner of their experiences. If children learned instead from observations of the parent's behaviours without knowing rewards, which in some cases, would be a more realistic assumption (Wu et al., 2022), the strength of selection for social learning would probably be weakened. We also assumed that the number of behaviours and their payoffs were constant within a given simulation, but this fails to account for evolutionary feedback which creates new behavioural opportunities as time progresses (Chimento et al., 2022), e.g. via niche construction (Smaldino & Richerson, 2012; Heras-Escribano, 2020) or cumulative cultural evolution (Smolla & Akçay, 2019; Derex & Mesoudi, 2020). Group structure and processes such as homophily and other group-level biases could inhibit the evolution of social learning since successful out-group teachers could be cut off from in-group learners (Golub & Jackson, 2012). We assumed that agents choose behaviours via a softmax decision rule, which is more cognitively sophisticated than most social-learning models and captures the adaptiveness of real human learning mechanisms. This modelling decision was essential for developing our understanding of how social and individual learning can combine to multiply the problem-solving capacity of our agents, although it is still a pale shadow of the true sophistication of human learning capabilities (Schulz et al., 2020; Wu et al., 2022a). More realistic and powerful individual learning could further support the evolution of social learning. These considerations provide novel opportunities to expand the evolutionary theory of social learning by adding such learning mechanisms to our model.

In our model, numerous behavioural options led agents to be more cognitively flexible via the softmax decision rule, and thus better able to adjust their behaviour if social learning provided outdated information. This explains why human social learning is widespread despite the fact that that the genus *Homo* emerged in, and continues to face, substantial non-stationary environmental variability (Antón et al., 2014; Levin, 2015): in our model, social learning is supported by the ability to adaptively combine socially and individually acquired information. Variable conditions and long lifespan have often obviated the possibility of genetic adaptation to most specific ecological circumstances, requiring instead the evolution of learning mechanisms for dealing with uncertainty. This has been demonstrated somewhat in previous models of social learning evolution, but these models relied on limited individual learning mechanisms that are furthermore mutually exclusive with social learning. This may prevent social learning from evolving under such high values of environmental variability as we observed here.

By carefully identifying and operationalizing different forms of uncertainty common in social-learning models, we developed a more thorough understanding of how uncertainty can favour social learning and how different forms of uncertainty interact to favour its evolution. By explicitly modelling a key individual-level learning mechanism shared across taxa, namely the ability to adjust behaviour to uncertainty and new information, we found that social learning could evolve even if it regularly provides outdated information. Our work, then, both advances the theory of social learning and could eventually be of practical use in identifying specific mechanisms underlying adaptations to uncertain environments.

## Data Availability

The data used to make the figures presented here is available via OSF (https://osf.io/t8exa/).

## Supplementary material

For supplementary material accompanying this paper visit https://doi.org/10.1017/ehs.2023.11. click here to view supplementary material

## Research transparency and reproducibility

Model and analysis code is publicly available on GitHub (https://github.com/mt-digital/UncMod). A guide to running the analyses is available on the GitHub repository. The code version used to generate the results presented here has been archived with a persistent identifier through Zenodo: https://doi.org/10.5281/zenodo.7873161.
